# Rupture du long extenseur du pouce secondaire à une fracture du radius distal réparée par une greffe tendineuse partielle à partir du long extenseur radial du carpe avec un bon résultat fonctionnel

**DOI:** 10.11604/pamj.2017.28.152.12362

**Published:** 2017-10-18

**Authors:** Mustafa Nkaoui, Amine El Yazidi

**Affiliations:** 1 Service de Chirurgie Orthopédique et de Traumatologie, CHU Ibn Sina, Rabat, Maroc; 2 Service de Chirurgie Orthopédique et de Traumatologie, Centre Hospitalier de Beauvais, France

**Keywords:** Radius distal, fracture, rupture tendineuse, greffe, Distal radius, fracture, tendon rupture, graft

## Abstract

We report the case of a 36-year old female patient injured in a fall from a horse. Radiological assessment showed closed comminuted fracture of the lower extremity of the left radius with extensive displacement (A & B). Initial management was based on open osteosynthesis with screwed anterior plate and nails (C) followed by functional rehabilitation. Patient's evolution was marked by the occurrence of secondary fracture of the extensor longus, clinically objectified by thumb extension deficit and confirmed by dynamic Ultrasound (D). The patient underwent surgery with exploration of the frayed tendon caught in a voluminous bony callus (E). Conservative treatment was not possible, repair was based on resection and partial graft from the extensor carpi radialis longus after dissection via the same approach. Outcome after rehabilitation was excellent (F) with gradual resumption of work from the second month.

## Image en médecine

Nous rapportons le cas d'une patiente âgée de 36 ans, victime d'un accident de sport à l'occasion d'une chute d'un cheval. Le bilan radiologique objectivait une fracture communitive fermée de l'extrémité inférieure du radius gauche avec déplacement important (A et B). La prise en charge initiale a consisté en une ostéosynthèse à foyer ouvert avec plaque antérieure vissée et des broches (C) suivie d'une rééducation fonctionnelle. L'évolution a été marquée par la survenue d'une rupture secondaire du long extenseur objectivé cliniquement par un déficit d'extension du pouce et confirmé par une échographie dynamique (D); la patiente a été repris chirurgicalement avec à l'exploration un tendon effiloché coincé dans un important cal osseux (E), le traitement conservateur était impossible, la réparation a consisté en une résection greffe partielle à partir du long extenseur radial du carpe après son dissection par la même voie d'abord. Le résultat après rééducation était excellent (F) avec reprise progressive du travail à partir du 2^ème^ mois.

**Figure 1:**
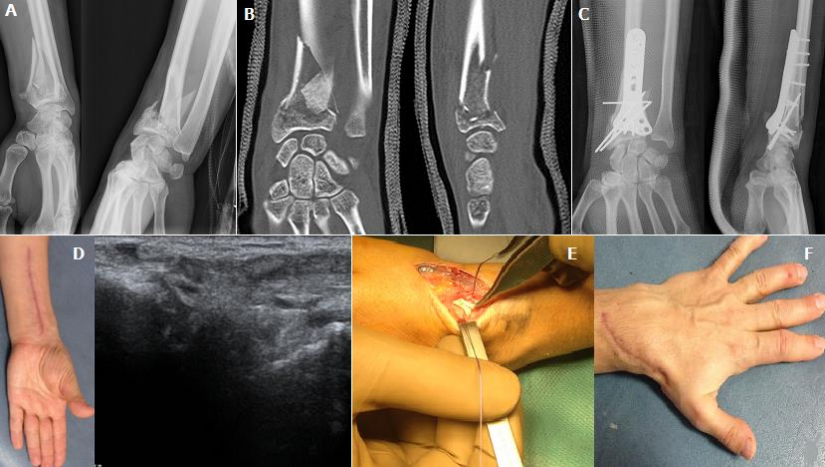
radiographie standard du poignet gauche montrant une fracture épiphysométaphysaire communitive déplacée de l’extrémité inférieure du radius; (B) scanner du poignet gauche objectivant la complexité de la fracture du radius distal atteignant la surface articulaire; (C) radiographie post opératoire de contrôle. Ostéosynthèse par plaque antérieure visée + broches avec une bonne réduction fracturaire; (D) aspect clinique et échographique de la rupture secondaire du long extenseur du pouce; (E) Image péropératoire. Aspect effiloché du tendon accroché dans un important cal osseux; (F) résultat clinique à j60 post opéraoire

